# A Novel Compound Heterozygous Mutation in TDRD9 Causes Oligozoospermia

**DOI:** 10.1007/s43032-024-01665-x

**Published:** 2024-08-22

**Authors:** Wenhua Wang, Yuming Feng, Jie Dong, Zheng Zhou, Jun Jing, Zixiong Li, Li Chen, Xiaoqi Lin, Jinzhao Ma, Bing Yao

**Affiliations:** 1 Center of Reproductive Medicine, Affiliated Jinling Hospital, Nanjing Medical University, Nanjing, 210002 Jiangsu China; 2 Center of Reproductive Medicine, Clinical School of Medical College, Nanjing Jinling Hospital, Nanjing University, Nanjing, 210002 Jiangsu China; 3 Department of Oncology, Nanjing Jinling Hospital of Nanjing University, Nanjing, 210002 China

**Keywords:** TDRD9, Compound heterozygous mutation, Oligozoospermia, Male infertility

## Abstract

Oligozoospermia is an important cause of male infertility for which treatment options are limited. Spermatogenesis is complex, and the causes of oligozoospermia remain largely unknown. Because genetic mutations are important factors of oligozoospermia pathogenesis, our study aimed to explore the genetic causes of oligozoospermia. Whole- exome sequencing (WES) was performed on one proband from a Chinese family who was diagnosed with oligozoospermia. The pathogenic mutations were confirmed by Sanger sequencing, and a minigene assay was used to determine the effect of the identified splicing mutation. We identified a novel compound heterozygous mutation in the TDRD9 gene, comprising a splicing mutation (c.1115 + 3A > G) and a frameshift mutation (c.958delC), in the proband; neither of these mutations were found in 50 unrelated healthy people. In addition, a minigene assay demonstrated that the frameshift produced partially truncated protein, and the splicing mutation led to a frameshift mutation and premature termination due to abnormal alternative splicing of TDRD9. These findings indicate that deleterious compound heterozygous mutation in TDRD9 could lead to oligozoospermia, highlighting the crucial role of TDRD9 in spermatogenesis and further clarifying the genetic causes of male infertility resulting from oligozoospermia. Our study expands the spectrum of TDRD9-related phenotypes and provides a new specific target for future genetic counseling.

**Supplementary Information:**

The online version contains supplementary material available at 10.1007/s43032-024-01665-x.

## Introduction

In recent years, the prevalence of infertility has increased significantly, with approximately 15% of couples worldwide suffering from infertility; male infertility alone or combined with female factors accounts for at least 50% of these cases. Common conditions that cause male infertility include azoospermia, oligozoospermia, and teratozoospermia. Oligozoospermia is a condition in which the sperm concentration falls below the reference limit of 15 million sperm per milliliter at ejaculation. Clinically, oligozoospermia can be caused by many conditions that either directly or indirectly damage the testicles, including congenital and hereditary disorders, hormonal disorders, and reproductive tract infections [1]. Numerous studies have demonstrated a strong genetic basis for oligozoospermia, with genetic abnormalities, such as abnormalities in chromosome number or structure, azoospermia factor region (AZF) deletion on the Y chromosome and cystic fibrosis transmembrane conduction regulator (CFTR) gene mutations, reported in men with otherwise unexplained oligozoospermia and azoospermia [2]. Moreover, previous studies have identified more than 400 genes that are specifically or potentially associated with fertility regulation while potentially contributing to the widespread genetic heterogeneity associated with dyszoospermia [3]. For example, mutations in *RPL10L* and *MAGEB* have been reported to cause oligozoospermia [4]. However, mutations in only a few genes have been shown to cause male infertility, and the candidate pathogenic genes for oligozoospermia still need to be studied further.

Tudor domain-containing protein 9 (TDRD9) is an RNA helicase that is highly expressed in germlines [5]. TDRD9 expression has been detected in mitotic spermatogonia, meiotic spermatocytes and haploid spermatids in the testis. In male infertility cases, TDRD9 has been reported to be involved in the silencing of long intersperm-1 retrotransposons, suggesting an association between TDRD9 mutations and nonobstructive azoospermia [6].

In our study, we screened a novel compound heterozygous mutation of TDRD9 in a Chinese patient with oligozoospermia (c.958delC, p. His 320Ilefs*28; c.1115 + 3A > G). Through an in vitro minigene assay, we found that a splicing mutation in TDRD9 (c.1115 + 3A > G) generated two mRNA products: one was consistent with the wild-type transcript, and the other retained a 19 bp sequence in intron 8 that led to the formation of a truncated protein, providing direct evidence for the occurrence of oligozoospermia in this patient.

## Materials and Methods

### Human Subjects

The patient was diagnosed with oligozoospermia and received treatment from the Department of Reproductive Medical Center of Jinling Hospital. Informed consent was obtained from all participants prior to the start of our study. This study was approved by the Research Ethics Committee of Jinling Hospital.

### Genetic Analysis

We used a TIANamp Blood DNA Kit (TIANGEN) to extract genomic DNA samples from the peripheral blood of patients and their families. Whole-exome sequencing (WES, Carrier Gene, Shanghai, China) was used to scan and identify the entire genome for variants and identify new rare TDRD9 variants compared with GRCH37/hg19. The DNA of the progenitor was analyzed via the Illumina HiSeq X10 platform. Several complementary online software programs were employed for pathogenicity prediction: varSEAK (https://varseak.bio), NNSPLICE version 0.9 (https://www.fruitfly.org/cgi-bin/seq_tools/splice.pl), mutation tester (https://www.mutationtaster.org), and combined annotation-dependent depletion (CADD, https://cadd.gs.washington.edu/). The Genome Aggregation Database (gnomAD, http://gnomad.broadinstitute.org) and Exome Aggregation Consortium (ExAC) Browser (http://exac.broadinstitute.org/) were used to determine the mutant allele frequency in the general population. Sanger sequencing was used to validate candidate variants for the patient's parents.

### Minigene Assay

The c.1115 + 3A > G variant was identified at the donor splicing site of intron 8. Owing to the large sizes(lengths) of introns 7 and 8, it was not possible to clone the full sequence of the region from exon 7 to 9 into minigene vectors. Therefore, we used 3 pairs of primers for PCR integration of exon 7, exon 8, and exon 9, and 200–300 bp before and after each exon. The integrated fragment was then cloned and inserted into the pMini-CopGFP minigene vector via homologous recombination. The mutant and wild-type plasmids were then transfected into 293 T cells. After incubation for 48 h, total RNA was extracted with TRIzol, and then reverse transcription polymerase chain reaction (RT‒PCR) and agarose gel electrophoresis were performed. RT‒PCR products were collected for Sanger sequencing. The primers used for the wild-type and mutant vectors and the primers used for RT‒PCR amplification are shown in Supplementary Table 1.

### Molecular Modeling

SWISS-MODEL software (https://swismodel.expasy.org) was used to analyze the structure of the TDRD9 mutant protein, and PyMOL software (http://www.pymol.org) (PDB ID: Q8NDG6) was used to visualize the structure of theTDRD9 protein.

## Results

### Clinical Characteristics

The patient was 29 years old and was diagnosed with oligozoospermia. No sperm were found during routine examination of the semen, after centrifugation at 4000 r/min, samples containing 15 sperm/50 µl could be obtained. The chromosome karyotype was normal, and no Y chromosome microdeletion was detected. The levels of follicle-stimulating hormone (FSH), luteinizing hormone (LH), pituitary prolactin (PRL), testosterone (T), estradiol (E2) and inhibin B (INHB) were examined and all found to be within the normal range (Supplementary Table 2).

### Two Novel Pathogenic Variants Were Identified in TDRD9

**Fig. 1:**
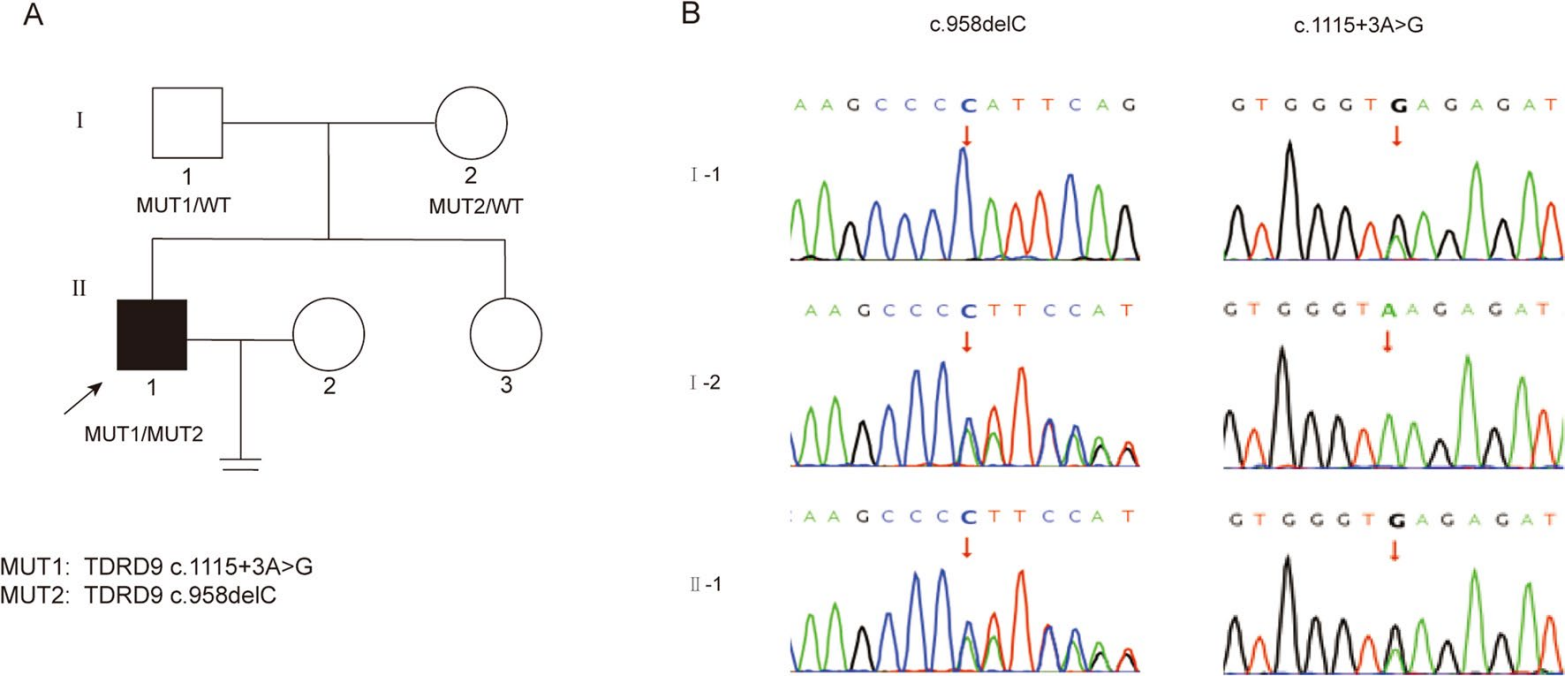
Genetic mapping and mutation identification of patients. **A**. The pedigrees of the family affected by TDRD9 mutations. Roman numerals denote generations, and Arabic numerals denote individuals. Squares represent the male, circular on behalf of female. Oligozoospermia patient is indicated by a black symbol with an arrow and normal individuals are indicated by open symbols. **B**. The heterozygous variants were verified by Sanger sequencing. The red arrows point to the mutated nucleotides, c.1115+3A>G heterozygous mutation was found in his father (I-1) and c.958delC heterozygous mutation was found in his mother (I-2)

Genomic DNA was extracted from peripheral blood samples from the proband (II-1) and his family members, and the likely pathogenic variants were detected via WES. The frameshift mutation c.958delC (NM_153046.3) located in exon 7 of TDRD9 was found in the proband (II-1). A splicing mutation, c.1115 + 3A > G (NM_153046.3), of TDRD9 was found at the splicing receptor site of intron 8 (Fig. 1A). The proband father (I-1) was a carrier of the splicing mutation c.1115 + 3A > G, and his mother (I-2) was a carrier of the frameshift mutation c.958delC (Fig. 1B), which suggesting that the mutations followed a recessive Mendelian inheritance pattern. Sanger sequencing confirmed the mutations in the proband and his parents. Table 1 shows an overview of the TDRD9 mutations.

**Table 1:** Overview of the TDRD9 mutations

Family	Individuals	Gender(age)	Location	Nucleic acid alteration	Variant allele	Protein alteration	Variant type
Family1	II-1	Male(29y)	Chr14:104,441,837	c.958delC	Heterozygous	p. His320Ilefs*28	Frameshift splicing
	II-1	Male(29y)	Chr14:104,452,660	c.1115 + 3A > G	Heterozygous	p. Asn373X^#^	

^**#**^**:** Two different mRNAs are produced, resulting in different protein products after translation. One encodes a truncated protein, while the other encodes a wild-type protein

### Prediction of the Effect of the Frameshift Mutation on the TDRD9 Protein

**Fig. 2:**
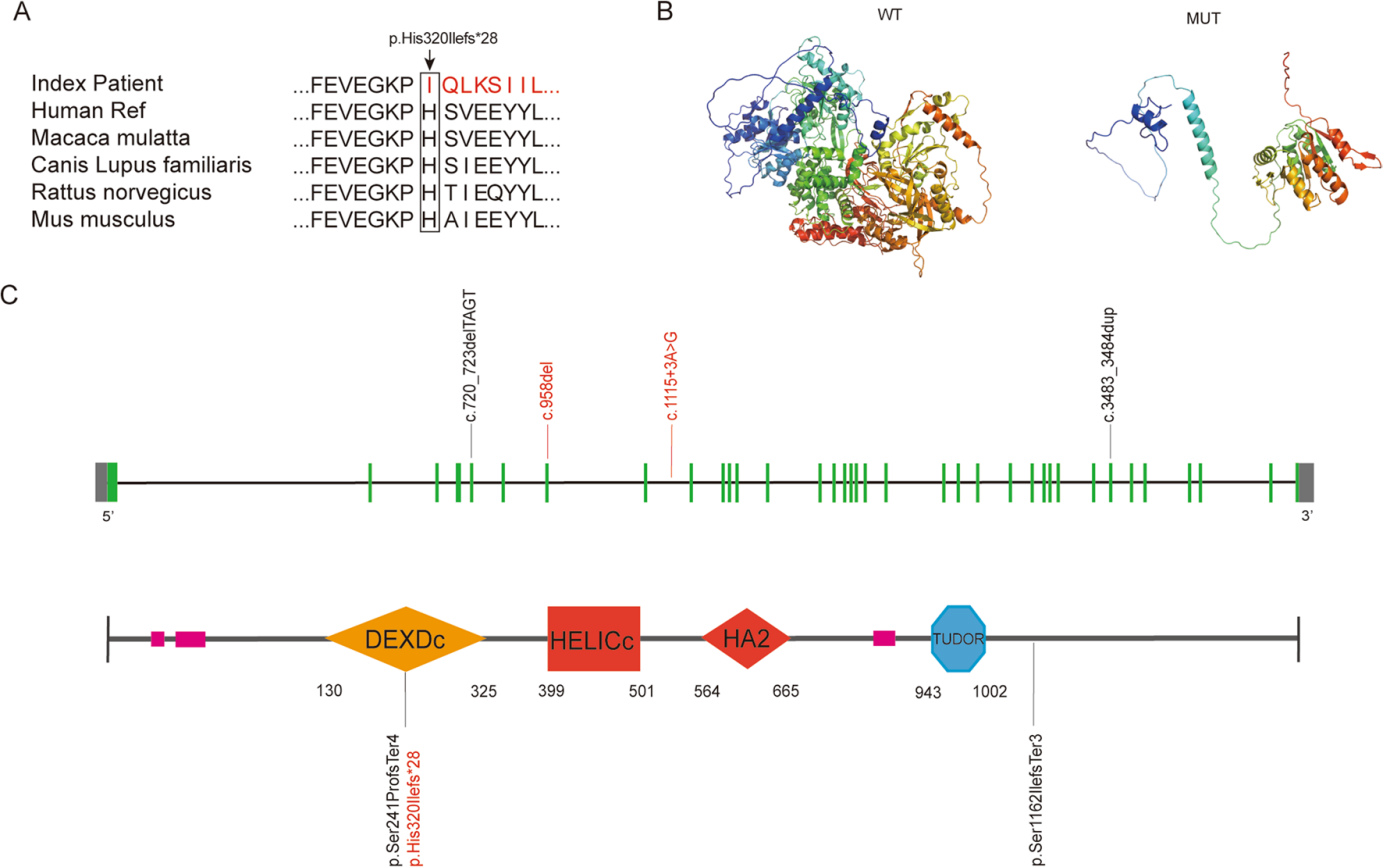
Prediction of the effect of TDRD9 mutations on this protein. **A**. Amino acid sequence comparison of TDRD9 protein in different species. **B**. The structural models of Wild-type and mutant TDRD9. **C.** The locations of the TDRD9 mutation. The coding area is represented by green boxes and the black line represents the intron. The location of the mutation in the structure of the TDRD9 protein is shown in the following section. Previously reported mutations are marked in black, and the new mutations identified in this study are marked in red

Analysis of amino acid residue conservation revealed that the damaged amino acid residues were highly evolutionarily conserved, suggesting that the mutation could be pathological (Fig. 2A). As shown in Fig. 2B, 3D structures of the wild-type and mutant TDRD9 proteins were created via SWISS-MODEL and rendered via PyMOL. The predicted 3D structure of the mutant protein was significantly disrupted compared with that of the wild-type protein. In addition, the mutation was predicted by Mutation Taster and CADD to be detrimental to the function of the TDRD9 protein (Table 2). These data suggest that this frameshift mutation (c.958elC) may be pathogenic. To confirm the specific association between this variant and oligozoospermia, we collected blood samples from 50 healthy individuals, extracted genomic DNA, and performed PCR. The PCR products were purified by gel electrophoresis and column chromatography and sequenced via Sanger sequencing. Th mutation was not found in the individuals without oligozoospermia. Figure 2C shows previously reported TDRD9 mutations associated with nonobstructive azoospermia as well as the new potentially pathogenic (causative of oligozoospermia) variants in our study.

**Table 2:** In silico analysis of c.958delC and c.1115 + 3A > G in TDRD9

Gene	Chromosome14	Mutation	Amino acid change	zygosity	gnomAD	EXAC	Mutation Taster_Score	Mutation Taster_ pred	CADD
TDRD9	104,441,837	c.958delC	p.His320Ilefs*28	Heterozygous	Not found	Not found	1	D	-
TDRD9	104,452,660	c.1115 + 3A > G	-	Heterozygous	Not found	Not found	0.99	D	16.49

### Minigene Assay to Evaluate the Potential Pathogenicity of the Splicing Mutation

**Fig. 3:**
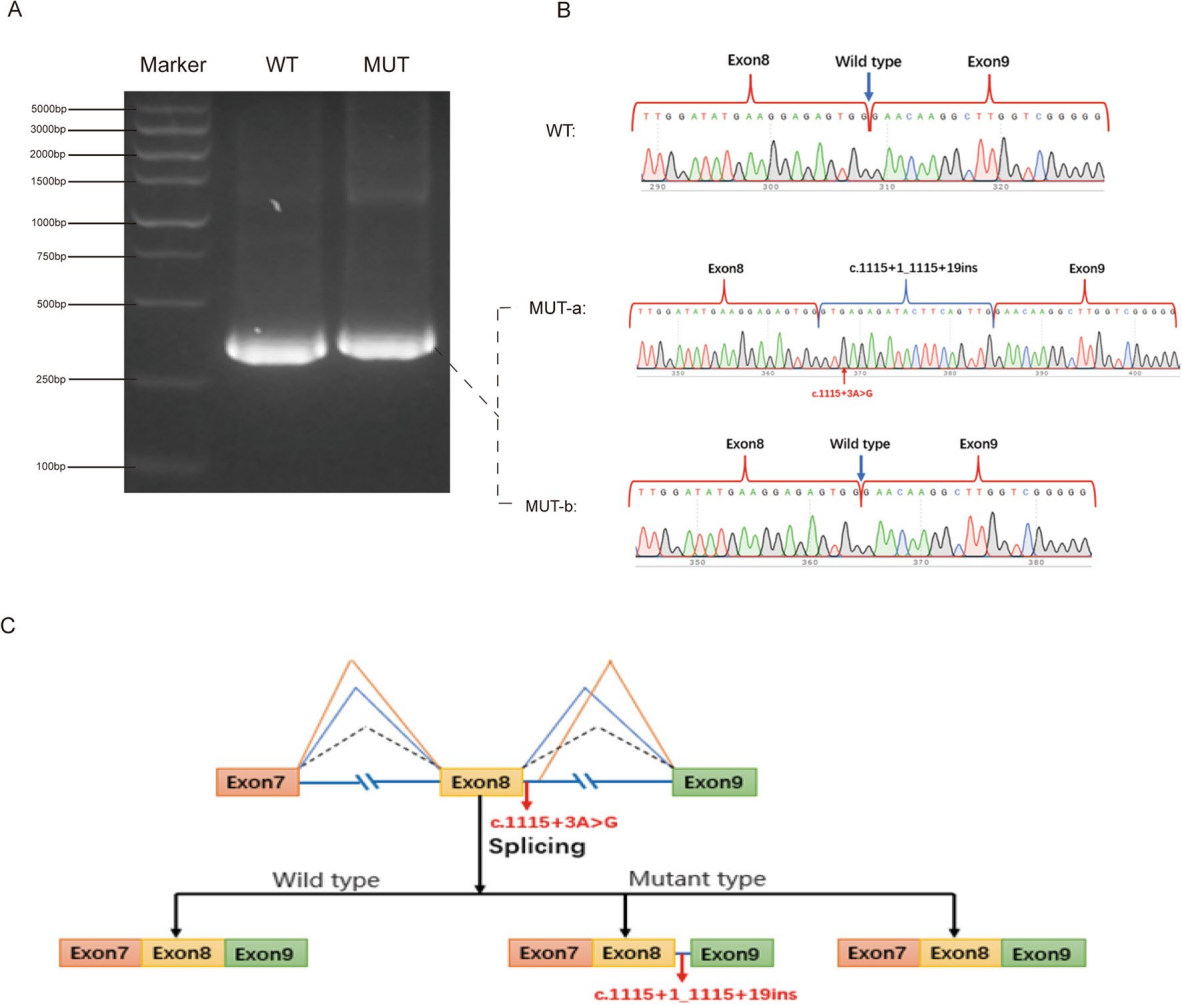
The effect of splicing mutation c.1115 + 3A > G of on TDRD9. **A**. Agarose gel electrophoresis showed that the TDRD9 mutation c.1115 + 3A > G had larger RT-PCR products than the wild type. **B.** Identification of aberrant splicing transcript with mutation. Sanger sequencing results showed that the mutation of TDRD9 splicing site led to two transcripts: (1) The retention of 19 bp sequence on the intron 8 of MUT-a, changing the original base sequence. (2) MUT-b: consistent with the wild type. **C**. A possible scheme to show the effect of TDRD9 c.1115 + 3A > G mutation. This mutation produces two conditions (1) that cause intron 8 to retain a sequence, resulting in protein truncation. (2) Consistent with the wild type

We utilized the online bioinformatics software varSEAK (Supplementary Fig. 1) and NNSPLICE version 0.9 (Table 3) to assess the effects of the c.1115 + 3 A > G variants. The results showed that TDRD9 mutation may lead to partial loss of function of the actual splicing site. We subsequently performed a minigene assay to determine the effect of the c.1115 + 3A > G mutation in TDRD9. Agarose gel electrophoresis revealed that the RT‒PCR product of this variant was larger than that of the wild-type product (Fig. 3A). Further Sanger sequencing revealed that the mutation resulted in two products: one containing intact exons 7, 8, and 9, identical to the wild-type transcript, and the other with partial retention of a 19 bp sequence in intron 8 (Fig. 3B-C), ultimately causing a frameshift and premature termination. We propose that the low level of normal transcript enables the patient to generate some sperm; however, the sperm count remains relatively low, resulting in oligozoospermia.

**Table 3:** Splice site prediction for wild-type and mutant TDRD9 by NNSPLICE 0.9 version

Donor site predictions for wild-type TDRD9				Donor site predictions for mutant TDRD9					
Start	End	Score	Exon	Intron	Start	End	Score	Exon	Intron
212	226	0.41	ctttgag**gt**gggggt		212	226	0.41	ctttgag**gt**gggggt	
519	533	0.98	actaatg**gt**gagagc		519	533	0.98	actaatg**gt**gagagc	
705	719	0.80	agagtgg**gt**aagaga		-	-	-	-	
796	810	0.64	taagaag**gt**ataact		796	810	0.64	taagaag**gt**ataact	
808	822	0.88	actatag**gt**actttt		808	822	0.88	actatag**gt**actttt	
847	861	1.00	aaatcag**gt**tagtat		847	861	1.00	aaatcag**gt**tagtat	
1285	1299	0.93	tcacaag**gt**caggag		1285	1299	0.93	tcacaag**gt**caggag	
1440	1454	0.50	ggttgca**gt**aagctg		1440	1454	0.50	ggttgca**gt**aagctg	
Acceptor site predictions for wild-type TDRD9				Acceptor site predictions for mutant TDRD9					
Start	End	Score	Intron	Exon	Start	End	Score	Intron	Exon
21	61	0.75	ttggtgtctttgctttaat**ag**acaattaaaagtagtagagg		21	61	0.75	ttggtgtctttgctttaat**ag**acaattaaaagtagtagagg	
587	627	0.99	tttttctttcactgttttt**ag**ctctctcctcatctcctgga		587	627	0.99	tttttctttcactgttttt**ag**ctctctcctcatctcctgga	
659	699	0.86	gttgctgtctctctcattc**ag**atgtttgatgacttggatat		659	699	0.86	gttgctgtctctctcattc**ag**atgtttgatgacttggatat	
809	849	0.41	ctataggtacttttttagt**ag**ttactgtatatattttaaaa		809	849	0.41	ctataggtacttttttagt**ag**ttactgtatatattttaaaa	
1103	1143	0.63	caatctccttctgtaatcc**ag**atgttcattcatttagctgg		1103	1143	0.63	caatctccttctgtaatcc**ag**atgttcattcatttagctgg

## Discussion

Male infertility, which affects approximately 7% of men, is a multifactorial pathological condition, and genetic factors are believed to account for at least 15%of cases. Approximately 2000 genes have been identified as involved in spermatogenesis, so the genetic landscape of this disease is complex [7]. The Tudor domain of Tudor domain-containing proteins (TDRDs) plays a crucial role in piRNA biogenesis and germ cell development. Twelve TDRDs have been identified in studies of humans and animal models, and defects in any of these TDRDs may interfere with spermatogenesis [8]. A previous study revealed that truncated TDRD1 was harmful to meiotic spermatocytes [9]. In 2018, a homozygous missense variant of the TDRD6 gene was considered the cause of oligoasthenoteratozoospermia (OAT), indicating that TDRD6 may be a new gene associated with OAT [10]. In 2023, another researcher used Tdrd6- deficient mice to further clarify the key role of TDRD6 in spermatogenesis and acrosome identification, and confirmed the causal relationship between TDRD6 variants and human OAT [11]. TDRD9 was also shown to be significantly expressed in the germ cells of both male and female mice. Specifically, immunohistology revealed that TDRD9 was present in haploid spermatids, meiotic spermatocytes (mostly at the pachytene stage), and mitotic spermatogonia in the testes [12]. This gene encodes an ATP-dependent RNA helicase that plays an important role in spermatogenesis. The TDRD9 (NM_153046.3) cDNA encompasses 4788 nucleotides, with a single open reading frame of 1382 amino acid (AA) residues. In turn, the TDRD9 protein contains several characterized structural domains, including the DEXDc domain between AA 130 and 325, the HELICS domain from AA 399 to 501, the HA2 domain from AA 564 to 665, and the Tudor domain from AA 943 to 1002.

Previous studies have shown that TDRD9 is essential for primary and secondary piRNA biogenesis, transposon inhibition, and male fertility. In mice, transposons are targets of the piRNA pathway, and their regulation is critical for spermatogenesis and male fertility [13]. Piwi proteins, characterized by their conservation and specificity within the Argonaute family towards the germline, are implicated in piRNA-mediated mechanisms, thereby participating in posttranscriptional silencing processes [14], and studies in the male mouse germline have indicated that the normal function of TDRD9 is necessary for the silencing of line-1 retrotransposons. Transposons have an important influence on genome structure and function, and the large class of TDRDs plays a key role in protecting germ cells from transposons [8]. The diminished expression of the TDRD9 gene observed in patients afflicted with cryptorchidism, particularly those exhibiting a heightened risk of infertility, suggests a potential association between gene instability resulting from compromised expression of transposon-silenced genes and the pathogenesis of azoospermia [15]. The first study to identify a homozygous deletion mutation in the human TDRD9 gene suggested that the mutation is associated with nonobstructive azoospermia [12].

Mutations in genes associated with spermatogenesis such as KLHL10, CDC14A, FANCM, and M1AP, reportedly cause oligozoospermia or severe oligozoospermia in humans [16]. In this study, we identified a novel compound heterozygous mutation in TDRD9, including one frameshift mutation, c.958delC, and one splicing mutation, c.1115 + 3A > G, in a Chinese patient from a nonconsanguineous family who was diagnosed with oligozoospermia. As predicted by CADD and Mutation Taster analysis(online), these two mutations may affect the structure or function of the protein. Among them, c.958delC replaces the histidine at position 320 with an isoleucine located in the DEAH/DEAD box helicase domain (DEXDc) domain, which is present in members of the DEAD-like helicases, a superfamily of multiple helicases that use ATP hydrolysis to unravel DNA and/or RNA [17]. This mutation leads to early termination of protein translation, thus truncating the Tudor domain, resulting in the failure of PIWI to bind normally to the Tudor domain, affecting PIWI-specific recognition and the piRNA-mediated transposon silencing process and thereby impacting male fertility [14]. The other mutation, c.1115 + 3A > G, is an intron variant located in intron 8. A minigene assay demonstrated that this mutation produces two alternative splicing isoforms, one of which exhibited an inserted of 19 bp, causing the asparagine encoded at position 373 of the protein to become a stop codon and form a truncated protein. The other transcript is identical to the wild type transcript, which is the reason why the patient still produces a small number of sperm. Therefore, we conclude that the c.958delC and c.1115 + 3A > G mutations in TDRD9 may contribute to male infertility. These findings broaden the spectrum of oligozoospermia-related TDRD9 mutations.

Our study has several limitations. First, only one case was included in the study; the associations between the c.958delC and c.1115 + 3A > G variants of the TDRD9 gene and oligozoospermia need to be validated in more patients. Second, oligozoospermia is a complex type of infertility characterized by gene–gene and gene-environment interactions. Therefore, future studies should include more genes associated with oligozoospermia to provide a more comprehensive genetic profile. Third, the functional relationship between TDRD9 variants in this study and oligozoospermia needs to be verified using animal experiments in mutant animal models. In the future, we plan to explore the link between TDRD9 mutations and oligozoospermia in more depth in order to more fully analyze the effects of TDRD9 mutations on spermatogenesis.

In summary, we identified a compound heterozygous mutation in TDRD9 (c.958delC, p. His 320Ilefs*28; c.1115 + 3A > G) in a Chinese male patient with oligozoospermia and confirmed that these two mutations are possible causative factors for oligozoospermia, leading to male infertility. Our clinical and genetic findings expand the understanding of the causative role of TDRD9 mutations in male-factor infertility.

## Supplementary Information

Below is the link to the electronic supplementary material.

Supplementary file 1 Supplementary Figure 1: Splice site prediction for wild-type and mutant TDRD9 by varSEAK. (DOCX 219 KB)

Supplementary file 2 Supplementary Table1: Primers for the minigene vectors construction and alternative splice sites detection in TDRD9. (DOCX 17 KB)

Supplementary file 3 Supplementary Table 2: The patient’s hormonal status. (DOCX 17 KB)

## Data Availability

The authors declare the availability of data upon request.
