# Supplementary figures and images for: A Novel Compound Heterozygous Mutation in TDRD9 Causes Oligozoospermia
Source: Reprod Sci. 2024 Aug 22;31(11):3413–9. doi: 10.1007/s43032-024-01665-x (PMC11527903; doi:10.1007/s43032-024-01665-x)

**Supplementary Figure1:** Splice site prediction for wild-type and mutant TDRD9 by varSEAK.


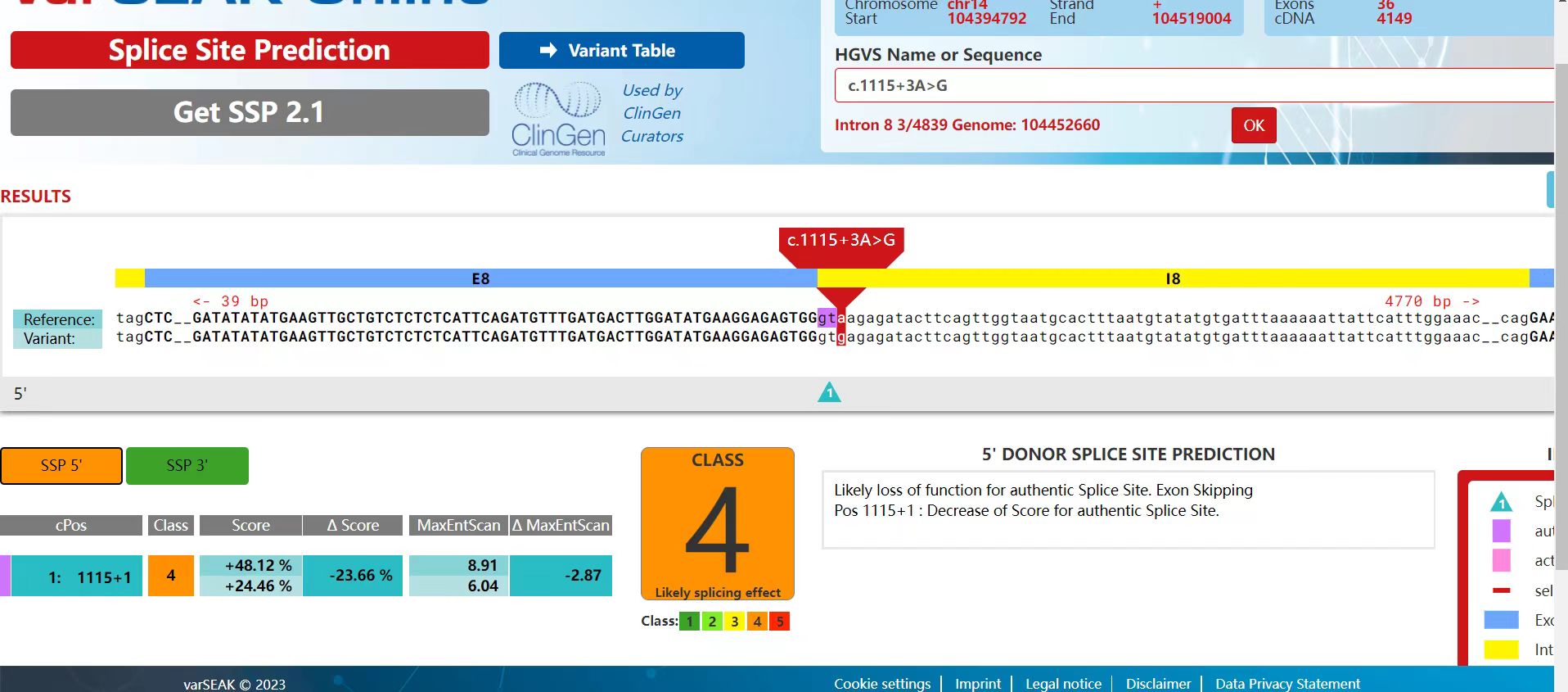

Supplement: Supplementary file 1 — Supplementary Figure 1: Splice site prediction for wild-type and mutant TDRD9 by varSEAK. (DOCX 219 KB) [file 43032_2024_1665_MOESM1_ESM.docx]
