# Supplementary material for: A Novel Compound Heterozygous Mutation in TDRD9 Causes Oligozoospermia
Source: Reprod Sci. 2024 Aug 22;31(11):3413–9. doi: 10.1007/s43032-024-01665-x (PMC11527903; doi:10.1007/s43032-024-01665-x)
Supplement: Supplementary file 2 — Supplementary Table1: Primers for the minigene vectors construction and alternative splice sites detection in TDRD9. (DOCX 17 KB) [file 43032_2024_1665_MOESM2_ESM.docx]

**Supplementary Table1:** Primers for the minigene vectors construction and alternative splice sites detection in TDRD9.

| Primer Name | Primer Sequence |
| --- | --- |
| TDRD9-AF | AAGCTTGGTACCGAGCTCGGATCCGTGGTCCTGATGTCGGCTACCATCAGCTG |
| TDRD9-AR | ATGTCTATGGGTTGGAGATGATGGATTTGCTAATTACCTTG |
| TDRD9-BF | CATCTCCAACCCATAGACATTTGGTGTGACTTAATCG |
| TDRD9-BR | GTTTTCCTTGGAGATGGAGTCTTGCTCTGTCACCAG |
| TDRD9-CF | ACTCCATCTCCAAGGAAAACTTCTGCAGATAGTAAGTTTTG |
| TDRD9-CR | TTAAACGGGCCCTCTAGACTCGAGCTGGCAAAAACACCAACACACTGCTTCG |
| TDRD9-MUT-F | GAGTGGGTgAGAGATACTTCAGTTGGTAATGCACTTT |
| TDRD9-MUT-R | GTATCTCTcACCCACTCTCCTTCATATCCAAGT |
| MiniRT-F | GGCTAACTAGAGAACCCACTGCTTA |
| TDRD9-RT-R | CTGGCAAAAACACCAACACACT |

Note: F, forward; R, reverse; AF/AR was a set of primers; BF/BR is a set of primers; CF/CR is a set of primers
