# Supplementary material for: A Novel Compound Heterozygous Mutation in TDRD9 Causes Oligozoospermia
Source: Reprod Sci. 2024 Aug 22;31(11):3413–9. doi: 10.1007/s43032-024-01665-x (PMC11527903; doi:10.1007/s43032-024-01665-x)
Supplement: Supplementary file 3 — Supplementary Table 2: The patient’s hormonal status. (DOCX 17 KB) [file 43032_2024_1665_MOESM3_ESM.docx]

**Supplementary Table 2:** The patient's hormonal status.

| Hormone name | Result | reference range | Unit |
| --- | --- | --- | --- |
| Follicle-stimulating hormone (FSH) | 8.88 | 1.4--18.1 | IU/L |
| Luteinizing Hormone (LH) | 5.21 | 1.1--8.7 | IU/L |
| Prolactin (PRL) | 208 | 44.5--375.24 | mIU/L |
| Testosterone (T) | 12.6 | 6.073--27.101 | nmol/l |
| Estradiol (E2) | 97 | <205.52 | pmol/l |
| Inhibin B(INHB) | 55.7 | 19.96--147.62 | pg/mL |
